# Multi-Targeted Mechanisms of Phytochemicals in Mitigating Cadmium-Induced Breast Cancer

**DOI:** 10.3390/medicines13020011

**Published:** 2026-03-24

**Authors:** Fidara F. Fidudusola, Caroline O. Odewumi, Lekan M. Latinwo, Oluwatobi A. Oguntunde, Samia S. Messeha, Karam F. A. Soliman

**Affiliations:** 1 College of Science and Technology, Florida A&M University, Tallahassee, FL 32307, USA; fidara1.fidudusola@famu.edu (F.F.F.); caroline.odewumi@famu.edu (C.O.O.); lekan.latinwo@famu.edu (L.M.L.); oluwatobi1.oguntunde@famu.edu (O.A.O.); 2 College of Pharmacy and Pharmaceutical Sciences, Institute of Public Health, Florida A&M University, Tallahassee, FL 32307, USA

**Keywords:** heavy metals, cadmium, breast cancer, phytochemicals, flavonoids

## Abstract

Cadmium (Cd) is an environmental toxicant originating from both natural processes and human activities. Cd has been strongly associated with multiple diseases, including breast cancer (BC). **Background/Objective**: Environmental Cd exposure represents a significant contributor to BC onset and progression. Cd-induced breast carcinogenesis is driven by a constellation of molecular events, including DNA damage, oxidative stress (OS), and the dysregulation of key signaling pathways. These include the ERK/JNK/p38 MAPK cascade, the PI3K/AKT/mTOR axis, NF κB activation, and Wnt signaling, all of which collectively promote tumor initiation, survival, and metastasis. This review underscores the complex interplay between Cd exposure and its effects on cancer-triggering factors. **Methods**: The complexity of the mechanisms Cd-induced BC, underlying Cd-induced BC makes it challenging to treat, highlighting the need for novel therapeutic strategies that complement or enhance conventional chemotherapy. Therefore, this review was developed by reviewing the literature and presenting the different aspects of the challenge associated with Cd exposure and BC therapy. **Results**: Phytochemicals, especially phenolics, alkaloids, carotenoids, terpenoids, and related plant-derived compounds, have emerged as promising candidates for mitigating Cd-induced BC. Their antioxidants, anti-estrogenic, and anti-inflammatory properties position them as potential chemopreventive and therapeutic agents capable of counteracting Cd’s molecular toxicity. **Conclusions**: The review presents current evidence linking Cd exposure to BC development and highlights the protective potential of selected phytochemicals in preventing or attenuating Cd-induced BC. Understanding these interactions reinforces the importance of phytochemical-based interventions as a strategy to reduce Cd-related cancer risk and support breast health.

## 1. Introduction

Most heavy metals are toxic compounds that the human body cannot tolerate, and their significant accumulation can be lethal [1,2]. Each heavy metal has a unique effect on the human body, and a serious disadvantage of heavy metals is that the body cannot metabolize them [2]. Certain heavy metals, such as iron (Fe), zinc (Zn), copper (Cu), and manganese (Mn), are essential to human survival at low concentrations; however, they can become toxic at higher concentrations. Other heavy metals, such as arsenic, lead, thallium, mercury, and Cd, are present in the environment and can eventually enter the human body. This group has no biological function, but it becomes hazardous once specific concentrations are accumulated [3].

Cd is a toxic heavy metal that enters the body through various sources, including contaminated air, food, and water, occupational exposure, car exhaust, and smoking [4,5]. Cd has been shown to have cytotoxic effects on various vital organs, including the breast [6]. As a metalloestrogen, Cd has estrogen-like characteristics that are linked to breast malignancy due to its interference with the normal mammary gland development [5,7,8]. A previous meta-analysis study highlighted the close association between Cd exposure and BC [9]. The incidence of BC was 1.13 times more common in women who had been exposed to higher concentrations of Cd [10]. Various epidemiological studies highlighted the impact of Cd exposure on the development of BC. These studies reported a high risk of BC among women exposed to Cd via environmental, occupational, and dietary exposure [11]. Long-term exposure to Cd can cause normal epithelial breast cells to undergo malignant transformation [12] and promote metastasis in patients with BC [13]. Notably, compared to normal breast tissue, malignant mammary gland tissue has substantially higher levels of Cd [14,15]. BC has emerged as a significant public health, societal, and economic issue. One of the most promising strategies for treating BC is the use of natural compounds as an adjunct to traditional BC treatment.

Naturally occurring flavonoids, such as quercetin, a class of polyphenolic compounds found in fruits, vegetables, and many plant-based products, have attracted significant interest because they can modulate oxidative damage and the related cellular signaling pathways controlling proliferation, death, and inflammation [16,17]. Furthermore, flavonoids trigger apoptotic cascades that control tumor development [18]. Recent in vitro and in vivo studies highlighted the significant role of consuming high-flavonoid-containing natural products in reversing Cd-induced epigenetic changes and oxidative imbalance [19]. Taken together, these investigations suggest that flavonoids may have a chemopreventive function, reducing the carcinogenic risks associated with Cd exposure and thereby emphasizing their potential in integrated approaches for cancer prevention.

## 2. Cadmium (Cd)

### 2.1. Cd Sources

Cd is classified as a toxicant with no known physiological advantages [20]. In 1817, Cd was initially found as an impurity in Zn carbonate (ZnCO_3_) by the German scientist Friedrich Stromeyer [21]. Cd is naturally found in the Earth’s crust at a concentration of 0.1 to 0.2 parts per million (ppm) [22]. Furthermore, industrial activities such as mining, smelting, and refining of zinc ore release substantial amounts of Cd [23,24] into the air, water, and food [24,25,26,27]. In the environment, Cd reacts with other elements to produce different significant chemical compounds such as Cd Oxide (CdO), a catalyst in redox reactions, hydrogenation reactions, polymerization, and cleavage, CdSO_4_ (used in batteries, pigments, and electroplating), and Cd (OH)_2_ (used in nickel-Cd batteries). Due to its economic value, Cd is widely used in industrial applications, including batteries, coatings, and plastic stabilizers. Unfortunately, nickel–Cd (Ni-Cd) batteries and Cd production present significant occupational health risks [28].

### 2.2. Cd Exposure and Excretion

**Figure 1:**
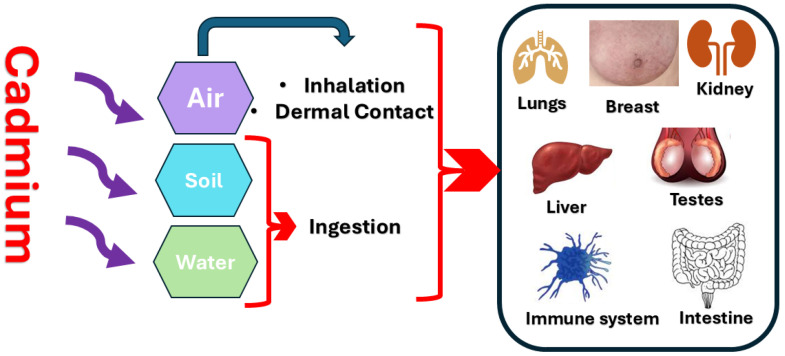
A summary of the sources of Cd exposure and its existence in different human body organs.

Cd can enter the human body mainly through three major routes: ingestion, inhalation, or dermal contact [29] (Figure 1). Apart from smoking and occupational exposure, ingestion is the main route for Cd exposure [30].

Dietary exposure to Cd is inevitable for humans and is typically ingested through contaminated food such as leafy vegetables, cereal, seafood, and water [31,32]. The level of Cd in plants has been linked to various environmental conditions, including soil, air, and water [33,34]. Lower concentrations of Cd are found in cereals, starchy roots, and vegetables. Rice grown in contaminated soil contains high levels of Cd. In Japan, where rice is the main staple, a condition known as ‘Itai-itai’ was evidently associated with the consumption of cadmium-contaminated rice [35]. A high level of this heavy metal is also found in particular species of aquatic animals, such as fish, crustaceans, scallops, oysters, and mussels, living in contaminated bodies of water [36,37]. Exposure via contaminated drinking water is primarily due to plumbing systems such as water heaters and coolers; however, this exposure is insignificant compared with dietary exposure [36]. Standards for oral Cd were set by various agencies, including the Agency for Toxic Substances and Disease Registry (ATSDR). In the United States, the estimated total dietary background value for Cd is 0.26 μg/kg/day, consisting of 0.18 μg/kg/day for food and 0.08 μg/kg/day for water [38,39]. The European Food Safety Authority (EFSA) established an acceptable intake of 0.36 μg/kg body weight/day [38,39].

Cd inhalation is the second pathway of exposure that occurs mainly through the respiratory tract [40]. This route of exposure could be occupational and/or non-occupational. Industrial workers and those living near industrial areas are at higher risk of inhaling Cd. Tobacco smoking is also a significant source of inhalation for smokers [41]. In comparison to non-smokers, smokers typically have more than twice as much Cd in their bodies and blood, since they are exposed to 1.7 µg of Cd in each cigarette [42].

Dermal exposure to Cd is less common; however, it can occur through contact with contaminated soil or water [43]. Cd absorption via the skin is considered modest, particularly when it enters the body as an inorganic metal. Dermal exposure can still be hazardous, particularly if the skin is scraped or abraded, or if there is ongoing contact with Cd-containing materials, such as batteries.

#### Excretion

In the biological system, Cd is excreted through urine [44], saliva [45,46], and breast milk [47]. Cd concentrations are highest in the kidneys and livers of mammals that consume diets high in Cd [48].

### 2.3. Cd Impacts on Human Health

Many epidemiological studies have demonstrated an association between chronic Cd exposure and the onset of various diseases, including brain, kidney, liver, lungs, bones, and reproductive diseases [32,40,49,50]. The functions of the central and peripheral nervous systems are severely impaired by Cd that enters brain cells, damaging proteins involved in neurotransmission. Cd reduces the activities of catalase (CAT), glutathione peroxidase (GPx), and superoxide dismutase (SOD), which leads to an abrupt increase in the free radicals and damage to DNA. These lead to behavioral abnormalities and a variety of neurological diseases, including multiple sclerosis, Alzheimer’s disease, and amyotrophic lateral sclerosis [51,52,53,54].

Renal tubular failure is another anticipated symptom of prolonged Cd exposure, resulting in lower serum levels of several biological molecules, including low-molecular-weight proteins, amino acids, glucose, parathyroid hormones, phosphorus, and calcium, which can lead to bone damage [55,56]. Cd-induced chronic renal diseases are a major cause of cardiovascular disease [55,57]. This negative impact elevates the levels of various biomarkers, including soluble suppression of tumorigenicity 2 (sST2), N-terminal pro-B-type natriuretic peptide (NT-proBNP), Growth Differentiation Factor-15 (GDF-15), and high-sensitivity cardiac troponin T (hs-cTnT) [58]. Furthermore, Cd impairs mitochondrial respiration and mechanisms controlling cellular metabolism and homeostasis [59]. A substantial correlation exists between acute Cd exposure and lung impairment. Olfactory function may be hampered by severe Cd inhalation that damages the nasal epithelium. Exposure to Cd levels greater than 5 mg/m^3^ may damage lung epithelial cells, which, in turn, contributes to the development of significant lung diseases in both humans and animals [60,61]. Moreover, Cd has strong teratogenic and mutagenic effects on both male and female reproductive systems. These epigenetic changes may impair placental function and fetal growth. Therefore, exposure to even low levels of this element can have a significant impact on fertility and pregnancy outcomes [62,63]. Having all these adverse health effects together could lead to the development of malignancy, as previously reported in many studies [64,65].

## 3. Cd and Cancer

Cancer has emerged as a significant public health, societal, and economic issue. Globally, cancer causes almost one in six deaths (16.8%). In the United States, it is the second-leading cause of death. In 2025, more than 2 million new cancer cases and over 600,000 cancer deaths are expected in the United States [66]. Cd induces cancer through various mechanisms, including inflammation, OS, ROS generation, epigenetic changes, inhibition of apoptosis, DNA damage, reduced DNA repair, alterations in gene expression, cell division, and abnormal DNA methylation [67]. The central aspect of Cd poisoning is OS. It has been demonstrated that mutagenesis and its effects on the cell cycle promote tumor growth. At the cellular level, the DNA repair system eliminates mutations arising from metabolism and environmental carcinogens; however, insufficient repair mechanisms allow damaged DNA to accumulate and worsen cancer. Cd inhibits DNA repair through this mechanism, which includes base and nucleotide excision repair, as well as mismatch repair. The lack of an effective DNA repair mechanism promotes the proliferation of damaged cells, which ultimately leads to mutations. Conversely, an effective DNA repair mechanism discourages the proliferation of damaged cells, thereby preventing mutations that may cause cancer [68].

Research has demonstrated that Cd may promote cell division by stimulating multiple proto-oncogenes, including c-fos, c-jun, and c-MYC, both In Vitro and In Vivo. These genes include the transcription factor AP-1 and several other genes related to cell division and growth [69,70]. According to epidemiological surveys, long-term exposure to Cd is strongly associated with an elevated risk of different tumors, including lung cancer, the most extensively documented, followed by breast and prostate cancer [71,72,73].

### Breast Cancer (BC)

BC is a crucial health concern among women due to its high mortality and morbidity rates. BC incidence has risen globally over recent decades, across both high- and low-income countries, and this rise is consistently linked to globalization [74]. In the United States, over 300,000 new female BC cases and over 42,000 deaths are anticipated. BC is a heterogeneous complex disease with varied etiology and pathological features. BC is usually called a group of diseases due to various biological subtypes with distinct molecular profiles and clinicopathological features [75]. According to WHO, the most recent histological classification is mainly invasive breast carcinoma of no special type (NST), which accounts for 70–80% of cases, special subtypes (lobular, tubular, cribriform, mucinous, metaplastic, papillary, and micropapillary), and precursor lesions; ductal carcinoma in situ, DCIS, and lobular in situ neoplasia, LIN [76,77]. The molecular subtypes of BC that have been identified by gene expression profiling are the receptor-positive (Luminal A, B, Normal-like, and HER-2—Human epidermal growth factor receptor 2 positives) and receptor-negative (TNBC—Triple-negative BC, or Basal-like) [78]. A significant percentage of BC cases are linked to various lifestyles, such as cigarette smoking, alcohol consumption, and obesity. In addition, other factors related to BC include hormonal therapy, pregnancy-related factors, inherited gene mutations (such as a BRCA1 mutation), age, and family history. Indeed, the likelihood of having BC is almost two times higher if a first-degree relative has had the disease, and potentially five times higher if the relative is diagnosed with BC at an early age [79]. The biological properties and molecular subtypes of BC affect how patients respond to treatment and how they behave clinically [80]. Even with adjuvant chemotherapy, the five-year survival rate for metastatic BC is less than 30% [81].

Although BC occurs in both sexes, male BC is a rare condition [82]. BC is more prevalent among certain racial groups, such as Israelis and Black African American men [83]. In the United States, 3000 incident cases and 500 deaths are reported for males, according to the 2025 statistical analysis data [66]. As with other cancers, the risk of male BC increases with age. These abnormal health conditions are associated with several factors, such as hormonal imbalances, obesity, Klinefelter’s syndrome, certain medications, and exogenous hormones (such as those used for gender reassignment) [84]. Furthermore, the BRCA2 gene is the most significant risk factor for male BC, with incidence rates as high as 10% in male BRCA2 carriers and a relative risk 80 times higher than that of the general population [85]. The metalloestrogenic property of Cd is the lead of BC development [86]. Chronic exposure to Cd was significantly linked to ER+ and HER-2 BC molecular subtypes [87], as well as the hormone receptor-positive lobular carcinoma histological subtype [88]. In TNBC cells, Cd promotes proliferation [89] and stimulates migration [90] and metastasis [91,92].

## 4. Mechanism of Cd-Induced BC

**Figure 2:**
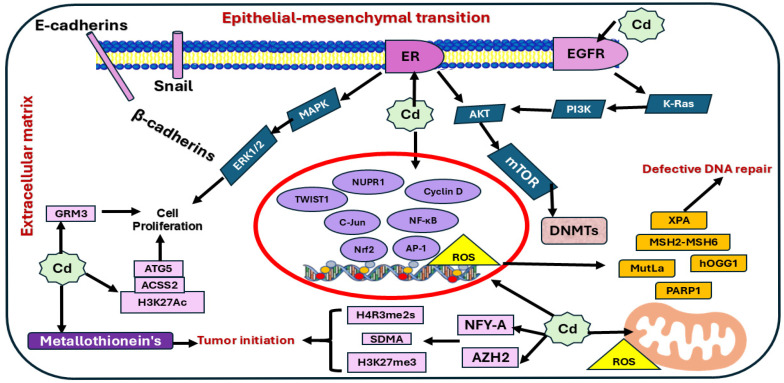
The possible interaction of Cd-stimulated signaling pathways in BC. The figure shows various signaling pathways that enhance cancer cell proliferation and metastasis: Extracellular signal-regulated kinase (ERK)/Jun N-terminal kinase (JNK)/P38 Mitogen-Activated Protein Kinases (p38MAPK), Phosphoinositide 3-kinase (PI3K)/AKT serine/threonine kinase (AKT)/mammalian target of rapamycin (mTOR), Nuclear factor kappa B (NF-κB), and Wnt signaling pathways.

**Figure 3:**
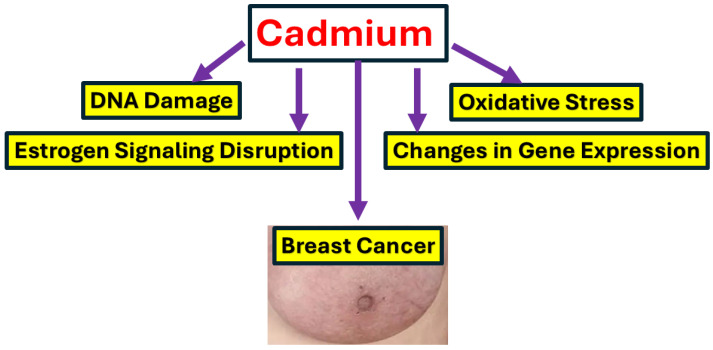
The possible mechanism of Cd-mediated BC initiation and progression. The metalloestrogen Cd acts through multiple mechanisms that contribute to BC development. Cd showed an ability to activate ERα, elucidates OS, DNA Damage, epigenetic changes, and impair DNA repair system.

The metalloestrogenic Cd acts through multiple mechanisms that contribute to BC development. Cd showed an ability to bind with ERα, inducing OS, DNA Damage, epigenetic changes, and impairing the repairing system (Figure 2 and Figure 3).

### 4.1. Cd and OS

The carcinogenicity of Cd is mediated by inflammation and OS induction, as demonstrated in various in vivo and in vitro studies [93]. Many metals, mainly Zn, Cu, Co (cobalt), Fe, and Se (selenium), are essential to the antioxidant mechanism. Unfortunately, Cd interacts with these metals, impairing their homeostasis and leading to OS [94]. OS is an imbalanced oxidant and pro-oxidant, as exhibited by increased free radical production. At the cellular level, OS may occur when reactive oxygen species (ROS) production exceeds antioxidant defenses. This mechanism can change the intracellular redox status [95]. Although Cd is not directly involved in the oxidation–reduction reactions that produce free radicals, it can indirectly generate ROS, such as superoxide, hydroxyl radicals, hydrogen peroxide, and nitric oxide. Cd showed a potential to attenuate the expression of various antioxidant enzymes, including catalase, superoxide dismutase (SOD)1/2, NAD(P)H quinone oxidoreductase (NQO), and glutathione S-transferase omega (GSTO)1; meanwhile, upregulated expression of inflammatory biomarkers including Interleukin 8 and 10, tumor necrosis factor alpha (TNFα), and Cyclooxygenase-2 (COX2) were demonstrated, along with increased ROS, and lipid peroxidation, leading to DNA damage [5], the events that worsened BC prognosis through drug-resistance and increased metastasis. Overexpression of COX-2 increases Prostaglandin E2 (PGE2) production, activating pathways [MAPK, Src, Akt, Vascular endothelial growth factor (VEGF), Hypoxia-inducible factor 1 alpha (HIF-1α)] that enhance BC progression. Therefore, non-steroidal anti-inflammatory drugs that target COX-2 can reduce BC risk. These inflammatory pathways modulate aromatase, which is crucial for estrogen production and thereby impacts BC development and progression [96]. In brief, inactivation of antioxidant enzymes, the displacement of redox-active metals, the depletion of antioxidants, and inhibition of the mitochondrial electron transport chain are the mechanisms of Cd-induced OS [68].

### 4.2. Cd Activates Signaling Pathways That Mediate Cancer

Previous studies have highlighted the potential of Cd to activate various pathways driving cancer, such as Extracellular signal-regulated kinase (ERK)/Jun N-terminal kinase (JNK)/P38 Mitogen-Activated Protein Kinases (p38MAPK), Phosphoinositide 3-kinase (PI3K)/AKT serine/threonine kinase (AKT)/mammalian target of rapamycin (mTOR), Nuclear factor kappa B (NF-κB), and Wnt signaling pathways [97].

MAPK (ERK, JNK, and p38 signaling pathways) control many cellular activities, such as cell differentiation, proliferation, migration, and apoptosis [98]. In BC, Cd activates the MAPK/ERK pathway by inhibiting protein phosphatases (PP2A, PP5), inducing ROS, MMP-2/9 expression, membrane-bound receptors, phosphate epidermal growth factor receptor (EGFR), Src kinase, and Erα [99]. Exposure to low concentrations of Cd was also found to activate MAPK through genetic changes, OS, or GPER signaling pathway in both ER+ and ER− BC [99,100].

Cd also activated the Nrf2 pathway by stabilizing the Nrf2 protein, increasing the cytoplasmic formation of the Nrf2/Keap1 complex, translocating the complex into the nucleus, and then disrupting the complex, which could support the adaptive response to OS [101].

PI3K/AKT/mTOR, the carcinogenesis initiator signal pathway [102] regulates cell growth, invasion, migration, and metastasis [103]. Cd activates these signaling pathways through triggering EGFR, G-protein-coupled receptor (GPCR), OS, ERα-dependent pathway, and damaging calcium homeostasis [100,104].

Abnormal activation of the Wnt pathway promotes the growth and renewal of cancer stem cells [105]. In MDA-MB-231 TNBC cells, Cd exposure activated integrin ꞵ1, Scr, Rac1, and FAK, inhibited GSK3ꞵ activity, upregulated β-catenin, and triggered T-cell factor/lymphoid enhancer factor (TCF/LEF)-linked transcription, the events that increased cell migration and metastasis [91].

NF-κB structural activation mediated chronic inflammation was previously shown to promote tumor growth by upregulating anti-apoptotic genes and fostering a microenvironment that supports cell survival [106,107]. Exposure to Cd activates NF-κB increases the downstream target genes, upregulates the expression of various oncogenes, like c-myc and c-fos, and the CAMP response element binding protein (CREB) [108,109]. Although the relationship between Cd and NF-κB has been the subject of numerous studies, further investigation is needed to determine how NF-κB contributes to Cd-induced carcinogenesis [97].

### 4.3. Mechanisms of Cd-Induced DNA Damage

Chronic exposure to Cd is strongly linked to increased DNA damage, reduced DNA repair, disruption of genomic stability and protein synthesis, the mechanisms implicated in Cd-induced carcinogenesis [97]. This process is mediated through multiple mechanisms, including the inhibition of tumor suppressor p53 binding to DNA, thereby impairing base excision repair (BER) [110], inhibition of DNA repair gene expression, downregulation of transcription factor activity, and functional interference with proteins via binding to zinc finger motifs [111]. Currently, evidence for Cd-mediated interference with double-strand DNA (dsDNA) repair pathways, such as homologous recombination repair (HRR) and non-homologous end joining (NHEJ), is still limited. Instead, most available data implicate Cd in the disruption of single-strand DNA (ssDNA) repair pathways, including BER, nucleotide excision repair (NER), and mismatch repair (MMR) [112]. Indeed, Cd inhibits various DNA repair pathways, including BER, NER, MMR, and NHEJ [113]. DNA repair enzymes are impacted by Cd exposure, the mechanism that promotes cancer onset [114]. Meanwhile, the transcriptional regulatory factor Special AT-Rich Sequence-Binding Protein 2 (SATB2) is upregulated, while other enzymes, including 8-oxo guanine DNA glycosylase (OGG1), all uracil glycosylases (hUNG), repair enzyme AP endonuclease (APE1), and DNA polymerases pol β and pol δ, are downregulated. Upon targeting these enzymes, the MMR system proteins MSH6 and MSH2, as well as NER initiators Xeroderma Pigmentosum Complementation Group C protein (XPC) and Xeroderma Pigmentosum Complementation Group A protein (XPA) are downregulated, which disrupts the NER pathway.

Cd can also interact with DNA through covalent binding, leading to the formation of bifunctional adducts, particularly within adenine–thymine (AT)-rich regions, and causing direct damage to guanine (G), adenine (A), and thymine (T) bases. Cd preferentially binds to the N3 and N7 positions of adenine and guanine, resulting in cleavage of DNA strands and disruption of glycosidic bonds. Although single-strand breaks (SSBs) account for approximately 90% of Cd-induced DNA lesions, Cd exposure is also associated with the induction of severe cellular double-strand breaks (DSBs) [115,116].

### 4.4. Changes in Gene Expression

Epigenetic changes are alterations in gene expression that occur independently of changes in the underlying DNA sequence. These changes include chromatin remodeling, histone tail modifications, DNA methylation, and the expression of long noncoding RNAs (lncRNAs) and microRNAs (miRNAs). In the presence of DNA methyltransferase and SAM (S-adenosyl methionine) as a methyl group donor, DNA methylation involves the covalent bonding of a methyl group to the cytosine to form 5-methylcytosine. Chromosome stability, gene transcription, and genomic imprinting are among the cellular processes that are regulated by DNA methylation. Post-translational covalent reactions involving the N- and C-terminal tails of H3 and H4 histones include methylation, acetylation, phosphorylation, Adenosine Diphosphate (ADP)-ribosylation, ubiquitination, and sumoylation, which impact the structure of the chromatin and the expression of genes [117]. miRNAs are small, non-coding molecules of 20–25 nucleotides that, depending on the degree of complementary base pairing, either degrade their target messenger RNA (mRNA) or prevent its translation. This process is involved in the post-transcriptional regulation of protein expression. While miRNAs are not translated into proteins, they are transcribed from DNA. The primary role of miRNAs is to suppress the expression of genes by disrupting the activities of mRNAs [118].

There is growing evidence in toxicogenomic research that exposure to heavy metals, such as Cd, is linked to epigenetic modifications [119]. Epigenetic change, particularly DNA methylation and noncoding RNA regulation, was suggested to be the primary mechanism by which Cd induces cancer [120]. Interestingly, prior research has demonstrated that Cd-induced DNA methylation alters the expression of genes linked to several types of cancer, such as liver, prostate, and lung cancer [121]. Additionally, it was discovered that Cd altered the expression of lncRNAs, and miRNAs mediated DNA repair, inflammation, and cell proliferation [122]. A recent study also revealed the Cd-induced epigenome in MCF-7 cells, indicating that Cd may have contributed to the development of BC by altering epigenetic changes [123].

Non-coding RNAs that regulate gene expression, such as microRNAs (miRNAs) and long non-coding RNAs (lncRNAs), are affected by Cd in terms of their expression and function [124]. Cd alters miRNA patterns associated with inflammation, stress responses, and cell survival, which can lead to conditions such as renal disease, osteoporosis, and cancer [125]. Epigenetic changes induced by Cd can activate oncogenes or silence tumor suppressor genes, thereby promoting cancer growth. Additionally, it affects reproductive health by altering genes linked to sex hormones and interfering with hormone signaling [126].

Immediate-early response genes (IEGs) like c-Fos, c-Jun, and c-MYC are activated by mitogenic stimuli and are often overexpressed in response to Cd exposure [69]. This overexpression is linked to Cd’s carcinogenic potential, as IEGs are commonly overexpressed in tumors and proliferating cells. Cd induces IEG overexpression in various cell lines, including those of rat and human origin, even at low concentrations. Overexpression can be transient or sustained, contributing to Cd’s role in cell transformation and cancer development [127].

### 4.5. Cd-Induced Estrogen Signaling Disruption

Many studies have reported that Cd induces endocrine-disruptive properties, characterized by estrogenic responses, following intraperitoneal injection [128,129,130,131]. These studies indicated that Cd could mimic estrogen activity, and it is considered a xenoestrogen [132]. It has been observed that Cd stimulates estrogen receptors (Erα) and promotes glandular cell growth [133]. Rationally, ER regulation can occur through either a genomic or a nongenomic pathway. As a xenoestrogen, Cd binds to ERα and prevents 17β-estradiol (E2) from binding, thereby promoting the proliferation of BC cells. In breast epithelial cells, genes controlling cell cycle, differentiation, and proliferation are regulated by estrogen via the estrogen receptor α (ERα) and estrogen receptor β (ERβ). Hence, cell proliferation is accelerated by Cd’s interaction with ERα’s hormone-binding domain. Hormonal resistance and the progression of cancer are other effects of Cd-induced ERα activation. G-protein-coupled receptor (GPR30), which binds E2 and initiates quick intracellular signaling pathways, is one of the nongenomic signaling pathways that estrogen promotes [134]. It has been demonstrated that Cd alters hormone receptor expression and contributes to the development of metabolic diseases. In various BC cell lines, Cd exhibits agonistic effects on G-protein-coupled estrogen receptor 1 (GPER) [135]. In the SKBR3 BC cell line, Cd promoted proliferation by activating cyclic adenosine 3′,5′-monophosphate (cAMP) synthesis and the ERK signaling cascade [134]. In contrast, this effect was significantly reduced in SKBR3 cells expressing the GPER-interfering mutant, suggesting it was GPER-dependent [136].

## 5. Phytochemicals and BC Treatment

**Figure 4:**
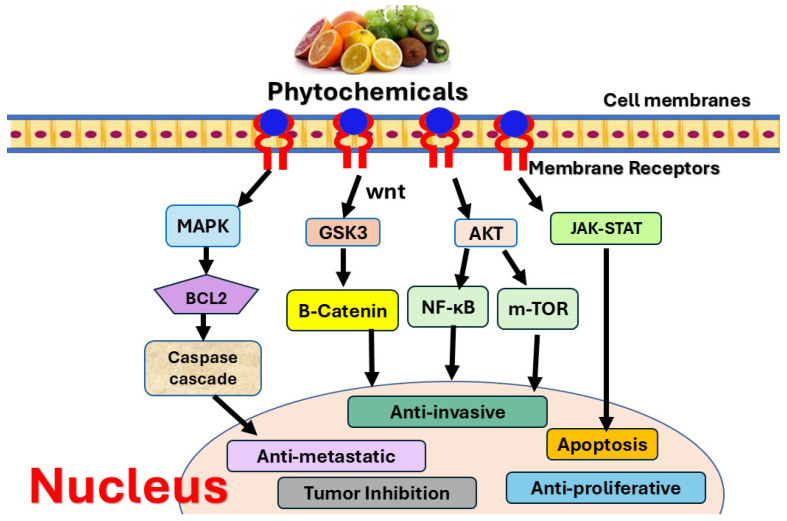
Phytochemicals modulate different signaling pathways in BC treatment. Phytochemical was previously found to trigger apoptosis, and induce anti-invasive, anti-proliferative, anti-metastatic effect that led to tumor inhibition. The figure shows various mechanisms employed by phytochemicals, including MAPK, AKT, JAK-STAT, Wnt, NF-κB, m-TOR, and GSK3 signal pathway.

Phytochemicals, natural bioactive compounds, have been extensively examined as anticancer agents due to their ability to modulate various oncogenic pathways involved in metastasis, such as angiogenesis, epigenetic alterations, cell cycle, apoptosis induction, immunomodulation, Epithelial-to-mesenchymal transition (EMT), and chemoresistance, among others (Figure 4). Phytochemicals are classified into different groups, mainly polyphenols, alkaloids, terpenoids, carotenoids, and organosulfur compounds [137,138].

### 5.1. Phenolics

Polyphenols include flavonoids, lignans, and stilbenes. This group is widely present in fruits and vegetables and characterized by antioxidants, anti-inflammatory, and chemopreventive properties, as shown in a previous in vitro study on Cd toxicity [139,140]. Polyphenols inhibit cancer metastasis by targeting biological markers, such as metalloproteinases and cytokines. Flavonoids target cancer cell survival by inducing apoptosis and disrupting cell cycle progression (Figure 4). In this review, we will focus on the most investigated compounds: quercetin, curcumin, genistein, and apigenin.

#### 5.1.1. Quercetin

Quercetin, a well-known flavanol, is an essential component of diets and supplements [125]. This compound is generously found in various plants, including buckwheat and bee pollen, as well as fruits, seeds, nuts, flowers, bark, and leaves. Glycosidic, the most common type of quercetin aglycone, is abundantly found in onions. Quercetin content is influenced by factors such as plant type, growth conditions, harvesting, and storage. For instance, storing foods at high or low temperatures can reduce the Quercetin concentration [141]. At the molecular level, quercetin targets multiple signaling pathways, including the Wnt/β-catenin, MAPK/ERK, and PI3K/Akt/mTOR pathways [142]. Quercetin has also been shown to exert anticancer effects by halting cell cycle progression, triggering apoptosis, inhibiting cell growth, modulating autophagy, and exerting anti-angiogenic and antimetastatic properties [143,144]. Quercetin acts as a potent antioxidant, scavenging ROS and maintaining oxidative balance by increasing glutathione (GSH) levels [145]. Additionally, it increases Nrf2 activity, which promotes the synthesis of many endogenous antioxidant enzymes and activates the antioxidant-responsive element (ARE) [146]. Numerous studies have revealed the synergistic effects of quercetin in combination with various medications such as doxorubicin, resveratrol, and catechin [147]. In BC research, Quercetin showed potential to inhibit cell proliferation in various models of BC, including MDA-MB-231, MDA-MB-468, MCF-7, and SK-BR-3 [148]. This mechanism was mediated by cell cycle arrest at the S/G1 Phase, upregulating proapoptotic markers such as caspases and downregulating anti-apoptotic markers. Quercetin also exhibited a synergistic effect with natural compounds (genistein, curcumin, and resveratrol) and chemotherapeutic agents (doxorubicin, cisplatin, paclitaxel, tamoxifen, and 5-fluorouracil), thereby decreasing treatment resistance [149,150,151,152]. The multi-targeted mechanisms of action of quercetin are a promising BC treatment; however, its clinical translation is challenging due to rapid metabolism and instability. This property urges comprehensive investigations to optimize various factors, such as safety and the effective dose, for effective treatment, particularly for BC patients [153].

#### 5.1.2. Curcumin

Curcumin, a polyphenol compound derived from the rhizomes of *Curcuma longa* [154], belongs to the yellow-colored curcuminoid group. This compound exhibits various biological properties, including anti-inflammatory, neuroprotective, immunomodulatory, and anticancer properties, by reducing ROS, phosphorylation, and activation of the MAPK signaling pathway [155]. Curcumin’s potential as a preventive and therapeutic drug has been investigated against multiple cancers, including BC, through targeting cancer stem cells linked to chemotherapy resistance and cancer recurrence [156]. In vitro studies using different BC cell models (MDA-MB-231, MCF-7, HCC1806, SK-BR-3) demonstrated that curcumin impacts crucial biochemical pathways involved in angiogenesis, metastasis, apoptosis, and cell proliferation [143,144]. These pathways include p53, PI3K/Akt, Wnt-β-catenin, NF-κB, JAK/STAT, and TGF-β pathways [157,158]. Curcumin inhibited Akt phosphorylation in both hormone-independent MDA-MB-231 cells and hormone-dependent MCF-7 cells in a time- and dose-dependent manner; however, MCF-7 cells were more sensitive to curcumin when combined with an Akt inhibitor simultaneously [159]. Furthermore, previous studies highlighted the cytotoxic effect of the compound in ER+/− BC cells [160]. The potential of curcumin to be a promising anticancer agent relies on its ability to augment the expression of many tumor suppressor proteins, such as p53, phosphatase and tensin homolog (PTEN), microRNAs (miR-15a/16/19/21/181b), whereas it inhibits EMT and various oncogenes (surviving, cyclin D1, Bcl-2) [161]. These mechanisms impact the PI3K/Akt signaling pathway and amplify its anticancer effects. In some BC cell lines, curcumin downregulated HER-2 and EGFR [162]. When used as an adjuvant therapy, curcumin can mitigate the adverse effects of radiation and chemotherapeutic agents (Doxorubicin, Paclitaxel, Cisplatin, 5-FU, Tamoxifen) and enhance its effectiveness [163]. In parallel with quercetin, reduced oral bioavailability is a significant challenge in BC clinical trials; however, the compound is safe with minor toxicity.

#### 5.1.3. Apigenin

Apigenin is a flavonoid that exhibits anticancer properties against various types of cancers, including BC, by modulating various signaling pathways mediating BC metastasis, such as MAPK/ERK, Wnt/β-catenin, PI3K/Akt, JAK/STAT, and NF-κB signaling pathways [164,165]. Although direct studies on Apigenin’s mitigation of Cd-induced OS in BC models are currently unavailable, multiple findings show that Apigenin can reduce OS by activating NRF2 and antioxidant enzymes [166], alter mitochondrial redox balance by attenuating CD38 [167]. Apigenin also induces apoptosis and modulates oxidative balance in human BC cells (MCF-7 and MDA-MB-231), and inhibits tumor growth via the PI3K/AKT/Nrf2 pathway in in vivo breast tumor models, implying that it may inhibit key pathways by which Cd promotes OS and cancer development [168].

#### 5.1.4. Genistein

The polyphenolic isoflavone genistein has been used in clinical trials as a promising anticancer compound by modulating crucial signaling pathways, including MAPK/ERK1/2, Wnt/β-catenin, NF-κB, and PI3K/Akt [169]. In BC models, Genistein has been proposed to alleviate oxidative damage induced by Cd via multiple signaling pathways that activate antioxidant genes [170].

### 5.2. Alkaloids

The anticancer effects of alkaloids have been demonstrated in various cancer cell models, and some (vinblastine, vincristine, and Taxol) have been approved by the Food and Drug Administration (FDA) as anticancer agents. Berberine and piperine alkaloids were also investigated in preclinical studies [171,172].

#### 5.2.1. Sanguinarine

Sanguinarine, the benzo-phenanthridine alkaloid, is the major compound in *Sanguinaria canadensis* plant rhizomes, which exhibits exceptional biological activity and anticancer properties [173]. Its anticancer effect is associated with DNA fragmentation and increasing cell death via intrinsic and extrinsic apoptotic pathways, as previously revealed in BC cells [174]. Sanguinarine induces apoptosis through various mechanisms, including free radical initiation and mitochondrial dysfunction, affecting proteins like signal transducer and activator of transcription 3 (STAT3), p53, B-cell lymphoma 2 (BCL2) family members, caspases, the inhibitor of apoptosis family (IAP), and extracellular signal-regulated kinase 1/2 (ERK1/2). It has been suggested that sanguinarine promotes apoptosis in the MDA-MB-231 BC cell line by up-regulating pro-apoptotic proteins and inhibiting anti-apoptotic proteins [175]. SANG can synergize with TNF-related apoptosis-inducing ligand (TRAIL)-linked apoptosis [176]. Also, in the MCF-7 BC cell line, combining SANG with a sub-lethal dosage of digitonin results in an enhanced cytotoxic effect [177].

#### 5.2.2. Catharanthus Roseus (Vinca Alkaloids and Terpenes)

Many therapeutic plants are found in the Indian community; one is *Catharanthus roseus* (*C. roseus*). This plant, also known as Vinca rosea or Madagascar periwinkle, has received special attention in phytoremediation for its ability to reduce the accumulation of heavy metals, such as Cd. *C. roseus* has been used in treating a wide range of diseases, including diabetes, wasp stings, bleeding, coughs, and cold symptoms [178]. *C. roseus* contains bioactive compounds, including terpenoids, flavonoids, and alkaloids, with chelating and antioxidant properties. This suggests they may protect against Cd-induced cytotoxicity by reducing cellular damage and OS [179]. Other properties of *C. roseus* extract include preserving serum waste materials, maintaining DNA integrity, protein profiles, and antioxidant enzyme levels, while also considerably reducing kidney and liver damage caused by Cd exposure. This plant contributes to increased lifespan, controls toxic side effects from chemotherapy, and improves healthcare systems [180].

Vinca alkaloids and terpenes were previously investigated in BC models (MCF-7, T47D, MDA-MB-231) [181]. These compounds showed potential to decrease cell viability, trigger apoptosis, halt cell migration, and invasion. Vinca alkaloids are considered safe adjunct natural alkaloids [182] that enhance chemotherapeutic agents (doxorubicin, cyclophosphamide, and prednisone) and reduce drug resistance [183], the major challenge in treating various types of malignancies, including BC [184,185,186]. These alkaloids use different mechanisms to target spindles, including disrupting microtubule dynamics, binding to tubulin, disrupting the cell cycle, and inducing apoptosis. Unfortunately, vinca alkaloids are linked to various side effects such as neurotoxicity, gastrointestinal complications, constipation, and liver dysfunction. On the other hand, terpenes exhibited low toxicity with minor effect on the liver, the properties that declare these compounds as non-carcinogenic in normal cells [187]. Still, clinical application of these compounds required more investigation to optimize dose and safety in BC patients.

### 5.3. Carotenoids

Carotenoids are extensively used for cancer prevention and treatment by controlling fibroblast activation and macrophage polarization [137], as previously demonstrated in various cancer research [188].

#### 5.3.1. β-Carotene

These natural carotenoids were found to target various markers linked to M2 macrophage (IL-6/STAT3 pathway) and fibroblast activation [189,190]. Furthermore, treating cancer cells with carotenoids suppresses invasiveness, migration, EMT, and cancer stem cell markers [190]. β-carotene derivatives such as all-trans retinoic acid (ATRA) can also inhibit cancer cell proliferation and trigger apoptosis [191]. Many clinical trials endorsed ATRA as a safe carotenoid for treating cancer patients, in particular when combined with various drugs (nanoparticle albumin-bound paclitaxel or gemcitabine) [192].

#### 5.3.2. Lycopene

Lycopene is another example of a carotenoid that manages many cancer-linked signaling pathways, such as NF-κB, p53, and SIRT1 [193]. Analogous to β-carotene, lycopene has shown a promising outcome in clinical trials by reducing cancer proliferation [194].

### 5.4. Terpenoids

Terpenoids, a class of phytochemicals, have shown anticancer effects against various cancer types, including BC.

#### 5.4.1. Nimbolide

Nimbolide is a known terpenoids that target malignant cells through various mechanisms, including apoptosis induction, upregulating the level of ROS, and moderate mTOR/ERK/PI3K/AKT oncogenic signaling pathways, which leads to a significant reduction in proliferation, migration, invasion, and metastasis of cancer cells [195,196,197,198].

#### 5.4.2. Ursolic Acid

Ursolic acid is another member of the terpenoid class that showed anticancer properties by altering many signaling pathways (p53, NF-κB, Wnt/β-catenin, and Ras), as well as targeting various proteins mediating apoptosis, such as tumor necrosis factor-related apoptosis-inducing ligand (TRAIL) and STAT3 [199].

#### 5.4.3. Withaferin A

In parallel with the above-mentioned examples, the terpenoid Withaferin A is considered an anti-proliferative terpenoid by halting cell cycle at G2/M phase and triggering apoptosis in many types of malignancies [200]. Withaferin A has potential therapeutic use in combating Cd-stimulated malignant changes in BC, as recent studies have shown that it can effectively decrease OS and reduce cancer cell viability in the presence of Cd by modulating mitochondrial function and ROS levels [201].

### 5.5. Other Natural Compounds

It is well established that Cd exposure causes OS and disrupts cellular homeostasis, ultimately activating oncogenic signaling pathways that promote BC [202,203]. Dietary flavonoids, such as hesperetin, naringenin, and epigallocatechin-3-gallate (EGCG), have shown promise as chemopreventive agents by counteracting these harmful effects. EGCG, the primary catechin in green tea, has demonstrated anticancer properties, primarily through the downregulation of oncogenic microRNAs, such as miR-25 [103,104], and the alteration of key pathways, including the PI3K/Akt/mTOR and p53/Bcl-2 signaling cascades, which collectively induce apoptosis in BC cells [204,205]. Citrus flavonoid naringenin has been shown to control several cellular processes, such as the FK506-binding protein 4 (FKBP4)/nuclear receptor subfamily 3 group C member 1 (NR3C1)/atomic factor erythroid 2–2-related factor 2 (NRF2) axis, which may help to restore the redox balance that was previously disrupted by Cd exposure [206]. Another bioactive flavonoid produced from citrus, hesperetin, enhances apoptotic responses in BC cells by triggering cell cycle arrest and senescence [204]. These pathways are characterized by their capacity to reduce OS- and Cd-related abnormal signaling, thereby hindering uncontrolled cell division and metastasis. The synergistic interactions among these natural compounds highlight their potential for incorporation into future therapeutic efforts and provide a multimodal strategy for counteracting Cd-mediated carcinogenic processes in BC.

## 6. Conclusions

Cd is a heavy metal that is found naturally in the Earth’s crust. However, its presence in the environment exceeds what nature produces due to various human activities. Cd exposure is implicated in BC onset through its estrogenic effects, disruption of DNA damage and repair mechanisms, OS, and alterations in gene expression. Natural compounds, especially flavonoids such as quercetin and curcumin, among others, offer a promising chemopreventive approach by mitigating the adverse effects of Cd and protecting breast tissue from changes that could lead to cancer. Effective dietary and therapeutic strategies for preventing BC urge further investigation into the mechanisms and effectiveness of these phytochemicals.

Many natural compounds, including phytochemicals, have low bioavailability and quick metabolism, making the practical translation of alternative therapy for Cd-induced BC extremely difficult. To overcome these obstacles, recent developments in nanotechnology, particularly nanoformulations, have proven effective [207]. The solubility, stability, and targeted distribution of anticancer drugs are improved by nanocarriers such as liposomes, solid lipid nanoparticles, and polymeric micelles, thereby increasing their bioavailability and therapeutic effectiveness [208]. For example, recent research demonstrates that using modified nanocarriers to co-deliver chemotherapeutic drugs such as doxorubicin with phytochemicals can alter several signaling pathways involved in cancer proliferation, thereby reducing effective doses and minimizing adverse side effects [209]. Additionally, the use of complementary combinations—standard chemotherapeutics combined with natural compounds—has been emphasized as an effective approach that leverages multiple modes of action to mitigate multidrug resistance, a prevalent obstacle in cancer treatment [210,211].

In conclusion, although exposure to environmental Cd is a challenging issue, an effective way to manage the risk and severity of BC is to introduce natural compounds with potential chemopreventive properties in our diets and therapeutic regimens. Therefore, future studies are warranted to elucidate the mechanisms underlying Cd-mediated BC and the role of natural compounds in mitigating this effect.

## Data Availability

No new data were created or analyzed in this study. Data sharing is not applicable to this article.
